# Peer Review of “COVID-19 and Cybersecurity: Finally, an Opportunity to Disrupt?”

**DOI:** 10.2196/29068

**Published:** 2021-05-06

**Keywords:** COVID-19, cybersecurity, challenges and disruption, data protection, privacy, health data

*This is a peer review submitted for the paper "COVID-19 and Cybersecurity: Finally, an Opportunity to Disrupt?"*

## Round 1 Review:

### General Comments

The contribution “COVID-19 and Cybersecurity: Finally, an Opportunity to Disrupt?” [1] is written with good language quality and is easy to understand. It is a Viewpoint on cybersecurity in COVID-19 times. Based on this topic, it is of interest for the journal.

Unfortunately, the contribution is not in the general JMIR style, which needs to be checked by the editor.

To summarize, there is nothing new in this article that has not been reflected in the general news media. Even for a Viewpoint, the technical references are completely missing. In the end, I do not see any value in this well-written but technically weak viewpoint.

### Specific Comments

#### Major Comments

1. The authors present their viewpoint, but all points are more or less mainstream. What is the “new message,” what is different from all the others?
2. Several points have technical backgrounds, which do not need to be described in a viewpoint article; however, the authors need to show that they are familiar with the literature in the mobile health cybersecurity domain. I do not see this point, and it needs strengthening.
3. When it comes to contact tracing apps, the presentation is rather weak, and several questions or facts are not resolved (eg, the distance estimation of tracing apps needs to be as correct as possible; otherwise, a lot of false positives or false negatives will plague this type of app). There are a lot of concepts in different countries for tracing apps; the authors do not describe these. In some countries, the reaction to a warning is left to the user, but what happens if larger groups disobey the warnings of tracing apps?
4. One argument has not been presented, namely that the increased use of electronic equipment in social distancing times increases the cybersecurity attack surface.
5. In the section on data sharing, the potential of false positives needs to be discussed, eg, technical limitations of distance estimations between different smartphone types should be critically reflected and a reference to this discussion should be given.
6. The authors do not reflect on the general cybersecurity risks of implemented mobile health apps (eg, transport security).
7. There are some reflections on difficulties for tracing apps, but they are not backed by references.
8. The title is very general, and the content does not fit the title; it needs to be less general and has to reflect that this is not a technical paper but a Viewpoint.

## Round 2 Review:

### General Comments

This paper is improved, but I am not convinced about the content, which is noncomprehensive and not novel (which has been well explained by the authors). There is no typical research method behind it, such as a scoping review or similar, which may be the reason that I do not like it. In my opinion, the world does not need such a viewpoint paper, but the editors have to decide.
